# 1,25(OH)_2_D_3_ Deficiency Induces Colon Inflammation via Secretion of Senescence-Associated Inflammatory Cytokines

**DOI:** 10.1371/journal.pone.0146426

**Published:** 2016-01-20

**Authors:** Yun Liu, Lulu Chen, Chunchun Zhi, Ming Shen, Weiwei Sun, Dengshun Miao, Xiaoqin Yuan

**Affiliations:** 1 Department of Anatomy, Histology and Embryology, Nanjing Medical University, Nanjing, China; 2 Institute of Stomatology, Nanjing Medical University, Nanjing, China; German Cancer Research Center, GERMANY

## Abstract

Epidemiological studies showed that 1,25-Dihydroxyvitamin D[1,25(OH)_2_D_3_] insufficiency appears to be associated with aging and colon cancer while underlying biological mechanisms remain largely unknown. Inflammatory bowel disease is one of the risk factors for colon cancer. In this study, we investigated whether 1,25(OH)_2_D_3_ deficiency has an impact on the colon of 25-hydroxyvitamin D 1α-hydroxylase knockout [Cyp27b1^−/−^] mice fed on a rescue diet (high calcium, phosphate, and lactose) from weaning to 10 months of age. We found that 1,25(OH)_2_D_3_ deficient mice displayed significant colon inflammation phenotypes including shortened colon length, thinned and disordered mucosal structure, and inflammatory cell infiltration. DNA damage, cellular senescence and the production of senescence-associated inflammatory cytokines were also increased significantly in the colon of Cyp27b1^−/−^mice. Furthermore, the levels of ROS in the colon were increased significantly, whereas the expression levels of antioxidative genes were down-regulated dramatically in the colon of Cyp27b1^−/−^mice. Taken together, our results demonstrated that 1,25(OH)_2_D_3_ deficiency could induce colon inflammation, which may result from increased oxidative stress and DNA damage, subsequently, induced cell senescence and overproduction of senescence-associated secretory factors. Therefore, our findings suggest that 1,25(OH)_2_D_3_ may play an important role in preventing the development and progression of colon inflammation and colon cancer.

## Introduction

Colorectal cancer is the third most commonly diagnosed cancer in USA and its incidence is higher in men than in women and significantly increases with age[1]; median age at diagnosis is about 70 years in developed countries[2]. Despite strong hereditary components, colonic inflammation is an important risk and progression factor of colon cancer. Chronic inflammation affects colon cancer development likely through the production of tumor-promoting cytokines, tumor cell invasive behavior, cellular proliferation and the promotion of angiogenesis[3]. Studies have shown that colorectal cancer is closely linked to diet and a so-called western lifestyle[4]. Vitamin D status of an individual is also influenced by both diet and lifestyle[5]. Epidemiological studies have consistently shown an inverse association between serum vitamin D concentrations and risk of colorectal cancer[6, 7]. Higher serum vitamin D levels also correlate with reduced risk for developing inflammatory diseases such as inflammatory bowel disease[8, 9]. A previous study has shown that Cyp27b1^-/-^ mice were more susceptible to DSS-induced colitis with increased IL-1 and IL-17 cytokines[10]. Vitamin D receptor (VDR) deficiency resulted in severe inflammation of the gastrointestinal tract in IL-10 KO mice which spontaneous develops colitis[11]. Vitamin D is a prohormone that can be metabolically converted from 25-hydroxyvitamin D by the enzyme 1α-hydroxylase [1α(OH)ase, encoded by Cyp27b1, to the active form, 1,25-dihydroxyvitamin D_3_[1,25(OH)_2_D_3_][12, 13]. Besides its function in the physiological regulation of Ca^2+^ and P_i_ transport and bone mineralization[14], 1,25(OH)_2_D_3_ plays multiple biological activities by binding VDR, a high-affinity nuclear receptor that alters the transcription target genes[5]. During the past decade, there are a number of evidence indicating an association between low levels of vitamin D and age associated diseases such as cognitive decline[15], osteoporosis[16], osteoarthritis[17], cardiovascular disease[18], hypertension[19], diabetes[20], and cancer[21]. As persons age, the risk for vitamin D deficiency significantly increases. The percent of older adults suffering from vitamin D deficiency ranges from 20 to 100% in the United States[22]. This indicates that vitamin D may play an important role in diseases that was associated with aging. Our previous data showed that disruption of Cyp27b1 gene in mice induced premature aging associated with multiple defects such as growth retardation, osteoporosis, hypophosphatemia, skin atrophy, temporomandibular joint (TMJ) osteoarthritis(OA), etc[17, 23]. These ageing-like symptoms of Cyp27b1^-/-^mice were similar to that of Klotho deficient mice. Studies showed that Klotho, an anti-aging gene, is regulated by vitamin D[24] which also supports the concept that vitamin D may have a function in ageing process.

It is generally accepted that cellular senescence is accompanied by a striking increase in the secretion of over 40 factors involved in intercellular signaling. This phenotype has been termed the “senescence-associated secretory phenotype”, or SASP[25]. SASP factors involving interleukins (IL-6, IL-8), growth factors (HGF), secreted proteases (MMP3) which turn senescent cells into pro-inflammatory cells can affect tissue microenvironments and stimulate tumor progression by promoting the proliferation and tumorigenesis of epithelial cells, stimulating angiogenesis, triggering an epithelial to mesenchymal transition, accelerating the invasion of transformed cells[26]. Cougnoux and his colleagues found that colibactin-producing *E*. *coli* enhanced colon tumor growth in both xenograft and AOM/DSS models by inducing the emergence of senescent cells secreting growth factors including HGF, FGF and GM-CSF, suggesting that SASP has a role in colon cancer development process[27].

We have previously shown that 1,25(OH)_2_D_3_ deficient (Cyp27b1^-/-^) mice fed with a “rescue” diet containing 2% calcium, 1.25% phosphorus, and 20% lactose to normalize the levels of serum calcium, phosphorus and parathyroid hormone(PTH), exhibited an erosive TMJ OA phenotype resulting from DNA damage, cellular senescence and production of senescence-associated inflammatory cytokines[17]. This suggests that 1,25(OH)_2_D_3_ deficiency could affect the disease progress by changing tissue microenviroment via inducing cellular senescence. In this study we investigated the influence of 1,25(OH)_2_D_3_ deficiency itself on colons by comparing the colon phenotype between Cyp27b1^-/-^ mice and their wild-type littermates maintained on the rescue diet from weaning to 10 months of age. We found that Cyp27b1^-/-^ mice developed colonic inflammation and that stromal cells of the lamina propria exhibited a senescence phenotype. The senescent cells accompanied by genotoxic damage led to SASP and some factors involved in SASP participated in the progression of colon cancer. These findings support the hypothesis that 1,25(OH)_2_D_3_ deficiency leads to colonic inflammation through secretion of senescence-associated inflammatory cytokines by senescent cells. These results implicate that insufficient 1,25(OH)_2_D_3_ may accelerate colon cancer progression by inducing colonic inflammation.

## Materials and Methods

### Mice and Genotyping

Cyp27b1^-/-^ mice were generated through breeding of heterozygous mice. The genotype of the mice was confirmed by PCR using mouse tail samples as previously described[23]. Wild-type littermates were used as control in all experiments. Animals were maintained on a 12-hour light/12-hour dark cycle under pathogen-free conditions. Male Cyp27b1^-/-^ and wild-type littermates of 8–10 months of age were used in this study. After weaning, they were fed with rescue diet (TD96348 Teklad, Madison, WI) containing 2% calcium, 1.25% phosphorus, and 20% lactose until 8–10 months old. We confirmed that serum calcium and phosphorus levels were normalized in the Cyp27b1^-/-^ mice and the littermates fed with the rescue diet. Animal welfare and experimental procedures were approved by the Institutional Animal Care and Use Committee of Nanjing Medical University (Approval ID 20111201).Genotyping was performed by PCR analyses of genomic DNA isolated from mouse tails. Genotyping forward and reverse primer sets were: 5'-GAAGTCCCTCCTGACACAGAAACCT-3' and 5'-CTCATAGAGTGTCCAGGAGAGCGTA-3'(cyp27b1), and 5'-ACAACAGACAATCGGCTGCTC-3' and 5'-CTCATAGAGTGTCCAGGAGAGCGTA-3'(neo).

### Tissue Collection and Analysis

Following euthanasia, full length of colon (from caecum to rectum) were removed and washed in PBS to remove fecal matter and then either: 1) fixed in 4% formalin and embedded in paraffin according to standard procedure and then paraffin-embedded tissues were cut 5 μm thick for HE staining and immunohistochemistry; 2) snap frozen in liquid nitrogen before RNA and protein extraction.

### Measurements of Serum Calcium, Phosphorus and 1,25(OH)_2_D_3_

Before mice were sacrificed, serum was obtained by retroorbital bleeding from anesthetized mice. Serum calcium and phosphorus levels were analyzed by an autoanalyzer (Beckman Synchron 67; Beckman Instruments). Serum 1,25(OH)_2_D_3_ level was measured by radioimmunoassay (DiagnosticProducts, Los Angeles, CA).

### Immunohistochemistry

Immunohistochemistry was performed on paraffin embedded colon tissues using specific antibodies (summarized in S1 Table). Dewaxed and rehydrated paraffin-embedded sections were incubated with methanol: hydrogen peroxide (1:10) to block endogenous peroxidase activity and then washed in Tris-buffered saline (pH 7.6). The slides were then incubated with the primary antibodies overnight at room temperature. After rinsing with Tris-buffered saline for 15 min, tissues were incubated with secondary antibody (biotinylated goat anti-rabbit IgG, Sigma). Sections were then washed and incubated with the Vectastain Elite ABC reagent (Vector Laboratories. Burlingame, CA) for 45 min. Staining was developed using 3, 3-diaminobenzidine (2.5 mg/ml) followed by counterstaining with Mayer's hematoxylin. 10 high-power fields (400× magnification) were randomly selected within each slide. Data are expressed as the average percentage of positive staining cells.

### β-Galactosidase (β-Gal) Staining

Pre-embedding β-Gal staining was performed following a modified version of a previously described method[28, 29]. Briefly, fresh colon tissues were removed and washed in PBS to remove fecal matter. Then the tissues were fixed in fix solution (2% paraformaldehyde and 0.2% glutaraldehyde in PBS) for 30 min on ice with shaking. Following fix, tissues were washed three times for 30 min in wash buffer (2 mM MgCl_2_, 0.01% sodium deoxycholate, 0.02% Nonidet-P40 (NP-40) in PBS) and incubated at 37°C in fresh senescence associated β-Gal (SA-β-Gal) staining solution (0.5 mg/ml 5-bromo-4-chloro-3-indolyl β-D-galactoside (X-Gal), 5 mM potassium ferrocyanide, 5 mM potassium ferricyanide in wash buffer) for 15hr with shaking and protection from light. After completion of staining, tissues were washed three times in PBS and embedded in paraffin according to standard procedure. Paraffin-embedded tissues were cut 5 μm thick and counterstained with nuclear fast red. 10 high-power fields (400× magnification) were randomly selected within each slide. Data are expressed as the average percentage of positive staining cells.

### RNA Isolation and Real-Time RT-PCR

RNA was isolated from frozen colon tissue with TRIzol reagent (Invitrogen, Carlsbad, CA) according to the manufacturer's protocol. Reverse transcription reactions were performed with the PrimeScript™ 1st Strand cDNA Synthesis Kit (Takara Bio,Shiga, Japan).To determine the relative expression of genes of interest, real-time PCR was performed using SYBR green reagents in an ABI 7300 sequence detector (Applied Biosystems, Foster City, CA). The sequences of PCR primers used were listed in Table 1. GAPDH gene was used as the internal control. Efficiency of amplification for all primers was validated by determining the slope of CT versus dilution series.

**Table 1:** Specific primers used for Q-PCR.

Genes	Primers	Sequences (5′–3′)
IL-1α	Forward	CGGGAGGAGACGACTCTAAAT
	Reverse	CACGAACAGTTGTGAATCTGAGA
IL-1β	Forward	GAAATGCCACCTTTTGACAGTG
	Reverse	CTGGATGCTCTCATCAGGACA
IL-6	Forward	CTGCAAGAGACTTCCATCCAG
	Reverse	AGTGGTATAGACAGGTCTGTTGG
MMP-3	Forward	TTAAAGACAGGCACTTTTGGCG
	Reverse	CCCTCGTATAGCCCAGAACT
MMP-13	Forward	TGTTTGCAGAGCACTACTTGAA
	Reverse	CCCCCGCATGAGCGAGAACACTGAC
CathepsinK	Forward	GAAGAAGACTCACCAGAAGCAG
	Reverse	TCCAGGTTATGGGCAGAGATT
IL-8	Forward	CCAAGGAAAACTGGGTGCAGAG
	Reverse	GGCACAGGGGAACAAGGACT
HGF	Forward	ATGTGGGGGACCAAACTTCTG
	Reverse	GGATGGCGACATGAAGCAG
SOD1	Forward	ATTACAGGATTAACTGAAGG
	Reverse	CAATGATGGAATGCTCTC
SOD2	Forward	GACCTGCCTTACGACTATG
	Reverse	GAAGAGCGACCTGAGTTG
Prdx3	Forward	GGTTGCTCGTCATGCAAGTG
	Reverse	CCACAGTATGTCTGTCAAACAGG
Txnrd1	Forward	CCCACTTGCCCCAACTGTT
	Reverse	GGGAGTGTCTTGGAGGGAC
Catalase:	Forward	AGCGACCAGATGAAGCAGTG
	Reverse	TCCGCTCTCTGTCAAAGTGTG
GCL	Forward	GGGGTGACGAGGTGGAGTA
	Reverse	GTTGGGGTTTGTCCTCTCCC
HO1	Forward	AAGCCGAGAATGCTGAGTTCA
	Reverse	GCCGTGTAGATATGGTACAAGGA
NQO1	Forward	AGGATGGGAGGTACTCGAATC
	Reverse	AGGCGTCCTTCCTTATATGCTA
Txn1	Forward	CATGCCGACCTTCCAGTTTTA
	Reverse	TTTCCTTGTTAGCACCGGAGA
Nrf2	Forward	TCTTGGAGTAAGTCGAGAAGTGT
	Reverse	GTTGAAACTGAGCGAAAAAGGC
Klotho	Forward	TGTTCTGCTGCGAGCTGTTAC
	Reverse	CCGGACTCACGTACTGTTTTT
GAPDH	Forward	TGGATTTGGACGCATTGGTC
	Reverse	TTTGCACTGGTACGTGTTGAT

### Western Blot Analysis

Lysates from colon tissues were separated on SDS-PAGE, transferred to polyvinylidene fluoride (PVDF) membranes and blotted with primary antibodies (summarized in S2 Table), followed by appropriate secondary antibodies and chemiluminescent detection.

### Intracellular ROS Analysis

For analysis of intracellular ROS, total colonic cells from 10-month-old mice were incubated with 5 mMdiacetyldichlorofluorescein (DCFDA) (Invitrogen) and placed in a shaker at 37°C for 30 min, followed immediately by flow cytometric analysis in a FACS calibur flow cytometer (BectonDickinson, Heidelberg, Germany)

### Oxidative Stress Levels

Colon tissues from 10-month mice were homogenized in cold saline. Homogenate (10%) was centrifuged at 4000 rpm at 4°C for 10 min. Supernatant was used for measurements of hydrogen peroxide (H_2_O_2_)(A064 H_2_O_2_ detection kit) and total superoxide dismutase (T-SOD) (A001-1SOD detection kit) Detection kits were from Nanjing Jiancheng Bioengineering Institute in China. All examinations were performed according to the manufacturer’s instructions.

### Statistical Analysis

All data are presented as Mean ± SEM. Student’s *t* test was used for comparisons. P < 0.05 was considered statistically significant.

## Results

### Effect of 1,25(OH)_2_D_3_ Deficiency on Serum Biochemistry

**Fig 1:**
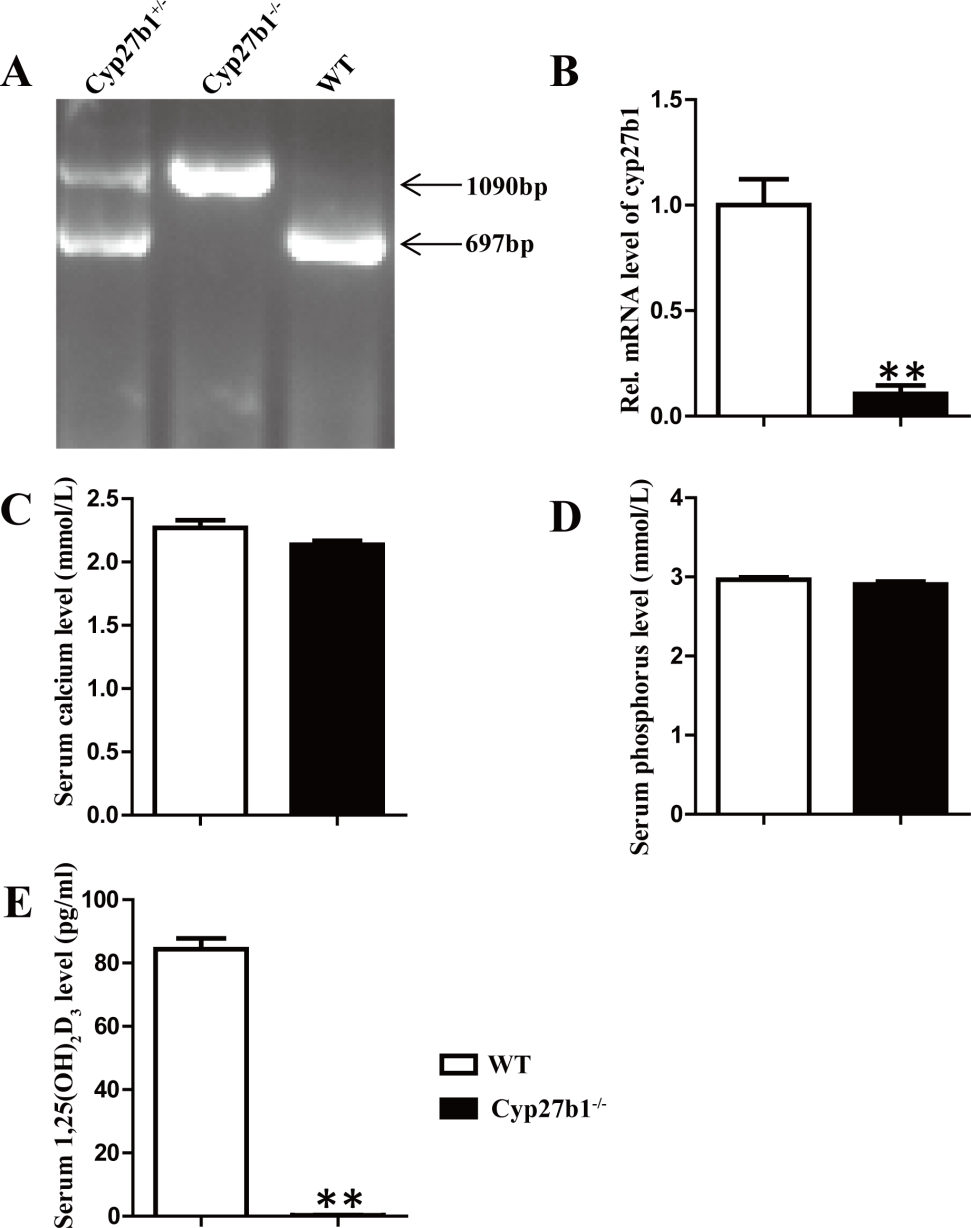
Effect of 1,25(OH)_2_D_3_ deficiency on serum biochemistry. (A) Deletion efficiency of the Cyp27b1 allele was analyzed using genomic DNA from the tail. (B) Quantitative PCR assay for Cyp27b1 mRNA in colons. (C) Serum calcium, (D) serum phosphorus and (E) serum 1,25(OH)_2_D_3_ from 10-month-old Cyp27b1^-/-^ and WT mice (n = 5; mean±SEM). **: p< 0.01.

Cyp27b1 knockout mice (Cyp27b1^-/-^) were identified by genotyping with genomic DNA (Fig 1A) and Cyp27b1 mRNA expression levels were dramatically lower in Cyp27b1^-/-^ mice than their wild-type littermates demonstrated by RT-qPCR (Fig 1B). In order to exclude possible effects of extracellular calcium and/or phosphorus and/or PTH, mice were fed on a rescue diet containing 2% calcium, 1.25% phosphorus, and 20% lactose from weaning to 10-month-old. We measured serum calcium and phosphorus levels and found no difference between WT and Cyp27b1^-/-^ mice (Fig 1C and 1D) whereas serum 1,25(OH)_2_D_3_ levels were undetectable in 10-month-old Cyp27b1^-/-^ mice (Fig 1E). Therefore, Cyp27b1^-/-^ mice had normal serum calcium and phosphorus levels after fed on the rescue diet and an effect of Cyp27b1 knockdown on colon may only result from 1,25(OH)_2_D_3_ deficiency itself.

### Effect of 1,25(OH)_2_D_3_ Deficiency on Colonic Inflammation

**Fig 2:**
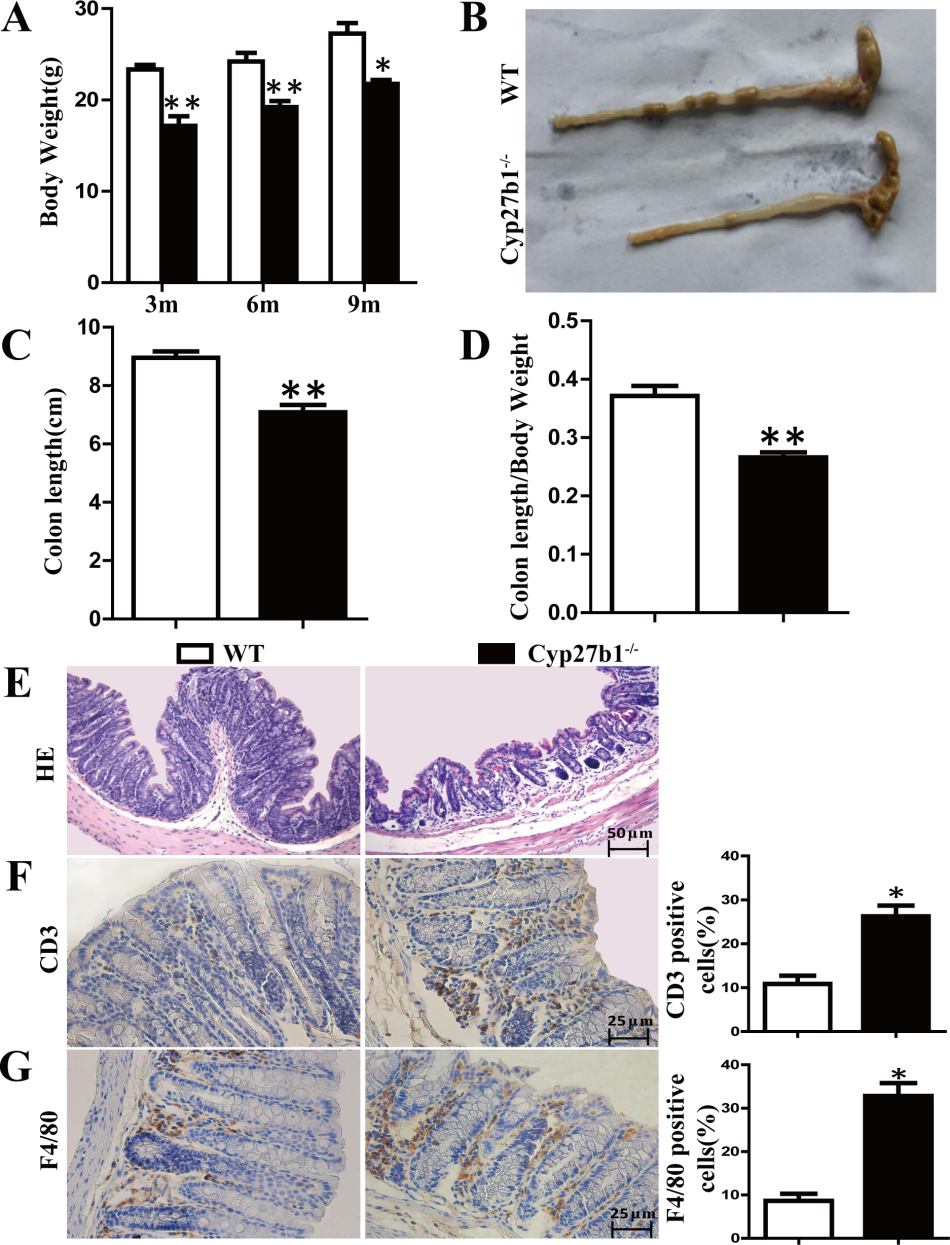
1,25(OH)_2_D_3_ deficiency induced chronic colonic inflammation. (A) Body weight from 3 month, 6 month and 10 month mice of Cyp27b1^-/-^ and WT. The full length of colon (B and C) and the ratio of length vs body weight (D) from WT and Cyp27b1^-/-^ mice with 10 month age. Values are mean ± SEM, n = 8. *P < 0.05, * * P < 0.01. (E) HE staining of colon section from WT and Cyp27b1^-/-^ mice. Magnification, ×200. Bar, 50μm. Immunohistochemical analyses of CD3 (F) and F4/80 (G) expression in colon tissues from the WT and Cyp27b1^-/-^ mice at 10 month. Magnification, ×400. Bar, 25μm. The histogram shows the mean percentage of the CD3 or F4/80 positive cells determined from five randomly selected fields. Magnification, ×400. Bar, 25μm. Values are mean ± SEM, n = 5. *P < 0.05.

To investigate whether 1,25(OH)_2_D_3_ deficiency resulted in colonic inflammation, we compared body weight, colon length and colon histologic structure between WT and Cyp27b1^-/-^ mice. Fig 2A showed that Cyp27b1^-/-^mice were significantly lighter than WT mice at all time points examined. Full colon length was shorter in Cyp27b1^-/-^ mice (Fig 2B and 2C) and the ratio of colon length vs body weight was also decreased in Cyp27b1^-/-^ mice (Fig 2D). Histologic results showed the thinner mucosa with disordered structure including crypt damage, decrease of goblet cells and submucosal edema in Cyp27b1^-/-^ mice (Fig 2E). Immunohistochemical studies further demonstrated that Cyp27b1^-/-^ mice had increased inflammatory cell infiltration compared with wild-type mice (Fig 2F and 2G, CD3^+^ T cells 26.25%versus 10.84%, P = 0.0105; F4/80^+^ cells: 32.82% versus 8.66%, P = 0.0176). These data suggest that 1,25(OH)_2_D_3_ deficiency lead to a colonic inflammation.

### Effect of 1,25(OH)_2_D_3_ Deficiency on DNA Damage and Cellular Senescence

**Fig 3:**
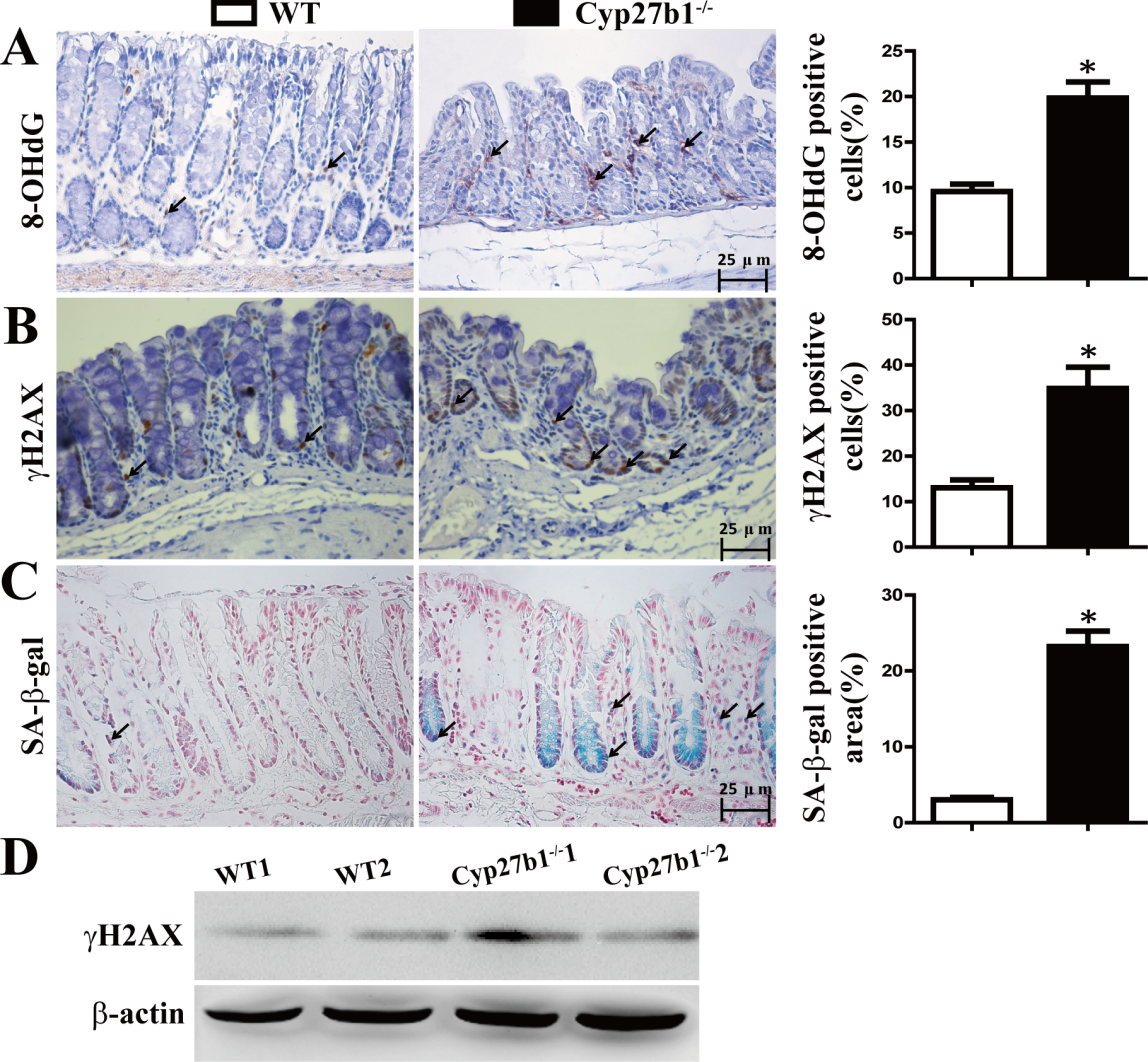
1,25(OH)_2_D_3_ deficiency induced DNA damage and cellular senescence. Immunohistochemical analyses of 8-OHdG (A) γH2AX (B) expression and analysis of SA-β-gal positive cells (C) in the colon from the WT and Cyp27b1^-/-^ mice. The positive cells in lamina proppria are marked by arrow. The histogram shows the mean percentage of the 8-OHdG or γH2AX, SA-β-gal positive cells determined from five randomly selected fields. Magnification, ×400. Bar, 25μm. Values are mean ± SEM, n = 5. *P < 0.05. (D): Western blot analyses of colonic γH2AX expression in the WT and Cyp27b1^-/-^ mice, β-actin was used as a loading control.

Our previous study has shown that 1,25(OH)_2_D_3_ deficient mice exhibited DNA damage and senesence in chondrocytes and osteoblasts[17]. In order to investigate whether this situation occurred in colon tissue, the DNA damage markers 8-hydroxyguanosine(8-OHdG) and γ-H2AX, were examined by immunohistochemical staining in paraffin sections of colon tissue from 10-month-old Cyp27b1^-/-^ and their wild-type littermates. β-Gal staining were also performed for checking SA-β-gal, the marker of cellular senescence. Results revealed that the percentages of 8-OHdG, γ-H2AX and SA-β-gal positive cells were increased significantly in Cyp27b1^-/-^ mice compared with wild-type mice (Fig 3A–3C). These positive cells were both localized in crypt and lamina propria. These observations imply that 1,25(OH)_2_D_3_ deficiency may induce DNA damage and cellular senescence of colonic epithelial and stromal cells.

### Effect of 1,25(OH)_2_D_3_ Deficiency on the Production of Senescence-Associated Inflammatory Cytokine

**Fig 4:**
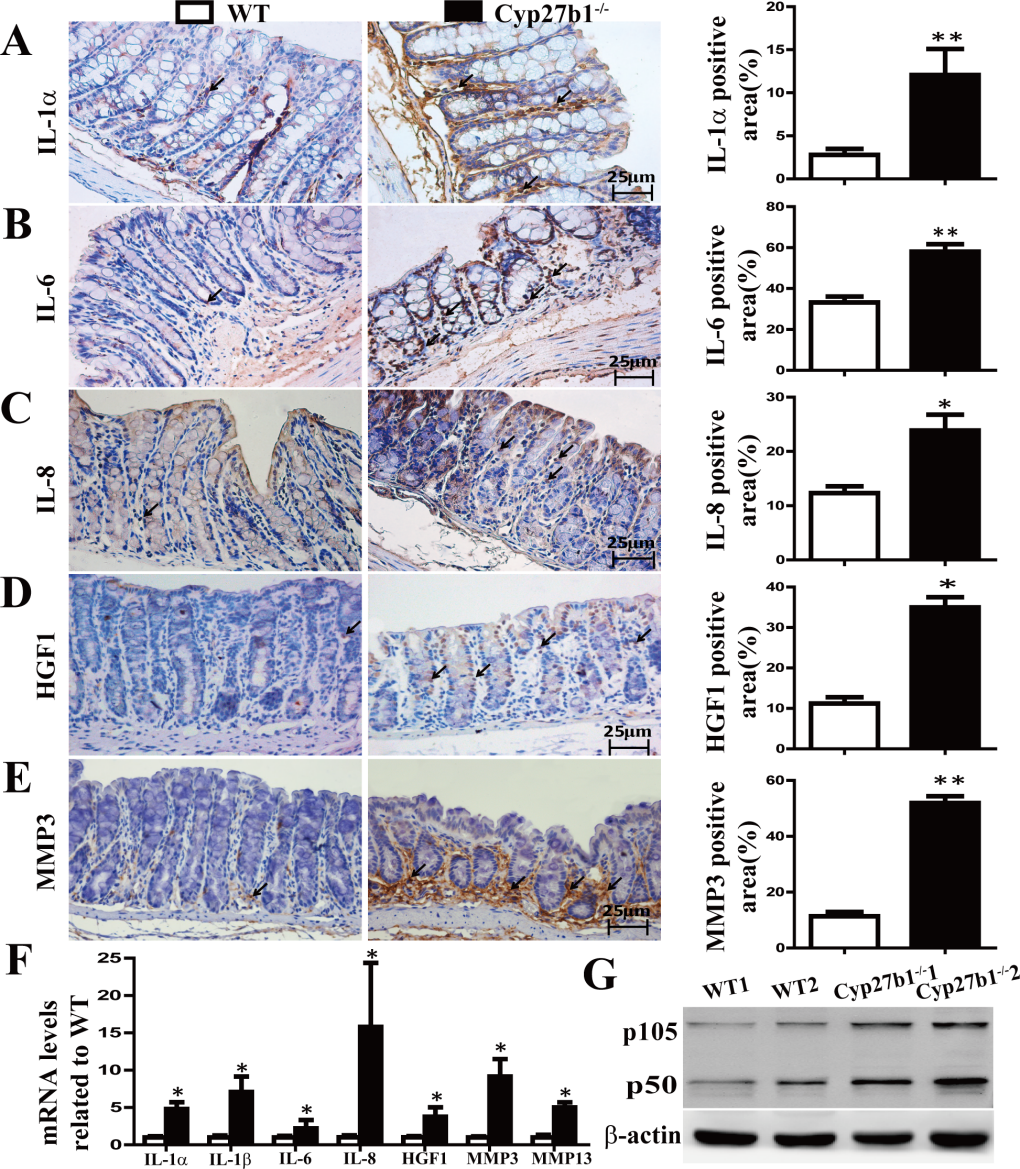
1,25(OH)_2_D_3_ deficiency induced the genes expression of the senescence-associated inflammatory cytokines. Immunohistochemical analyses of IL-1ɑ (A) IL-6 (B) IL-8 (C) HGF1 (D) MMP3 (E) expressions in the colon from the WT and Cyp27b1^-/-^ mice. Positive cells in lamina propria were labeled by arrow. The histogram shows the mean percentage of the positive area determined from five randomly selected fields. Magnification, ×400. Bar, 25μm. Values are mean ± SEM, n = 5. *P < 0.05, ** P < 0.01. (F) Real-time RT-PCR was performed on extracts of colon from WT and Cyp27b1^-/-^ mice for the gene expression of IL-1α, IL-1β, IL-6, IL-8, HGF1, MMP-3 and MMP-13. (G) Western blot analyses of colonic NF-κB (p105 and p50) expression in the WT and Cyp27b1^-/-^ mice, β-actin was used as a loading control.

Since senescent cells with persistent DNA damage secrete senescence-associated inflammatory cytokines, we sought to investigate whether these cytokines were secreted in 1,25(OH)_2_D_3_ deficiency mice. As shown in Fig 4F, the gene expression levels of IL-1α, IL-1β, IL-6, IL-8, HGF1,MMP-3 and MMP13 were all up-regulated significantly in colon from Cyp27b1^-/-^ mice when compared with the wild-type mice. Immunohistochemical staining showed that there were significantly more IL-1α, IL-6, IL-8, HGF1, MMP-3 positive cells observed in colon sections of the Cyp27b1^-/-^ mice than those of wild-type mice (Fig 4A–4E).Since nuclear factor-κB (NF-κB) was found to be a master regulator of SASP[30]. We further measured the expression of NF-κB-p105/p50 by immunoblotting and found that NF-κB-p105/p50 expression levels in colon were up-regulated in Cyp27b1^-/-^ mice relative to WT mice (Fig 4G).These observations implicate that 1,25(OH)_2_D_3_ deficiency-induced colonic inflammation may result from overproduction of senescence-associated inflammatory cytokines.

### Effect of 1,25(OH)_2_D_3_ Deficiency on Redox Balance

**Fig 5:**
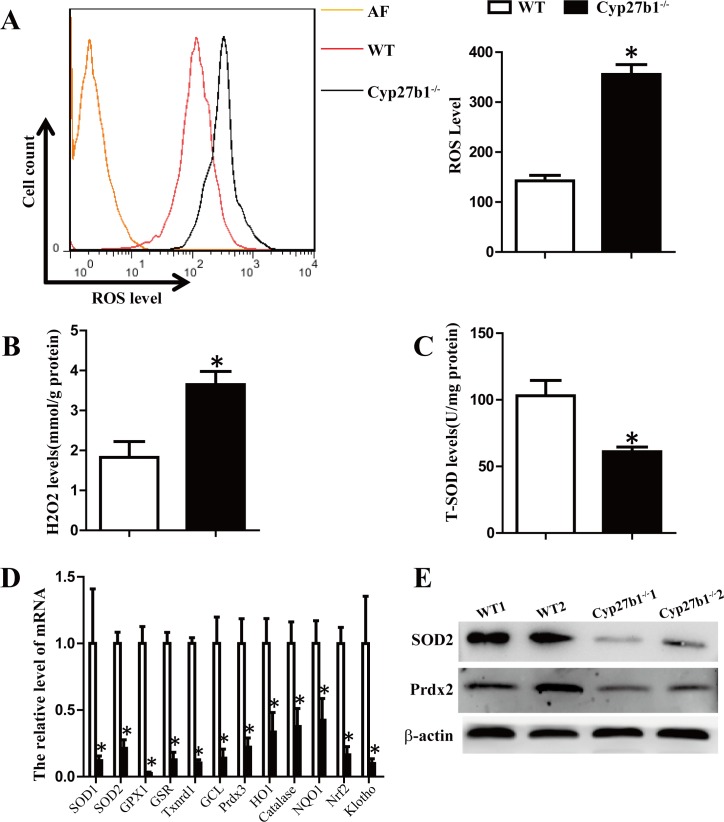
1,25(OH)_2_D_3_ deficiency induced the ROS production and decreased antioxidants. (A) Flow cytometry analysis of ROS in colons from the three groups of 10-month-old mice. (B) H_2_O_2_ and (C) Total SOD level of colons measured by spectrophotometry. (D)Real-time RT-PCR was performed on extracts of colon from WT and Cyp27b1^-/-^ mice for the gene expression of antioxidants. (E) Western blot analyses of colonic SOD2 and Prdx2 expression in the WT and Cyp27b1^-/-^ mice, β-actin was used as a loading control.

Imbalances between ROS and antioxidants can lead to oxidative stress that induces DNA damage response. Increased ROS and H_2_O_2_ levels were observed in colon tissue from Cyp27b1^-/-^ mice compared to wild-type mice (Fig 5A and 5B).We also measured the important antioxidant of SOD and found the total colonic SOD in Cyp27b1^-/-^ mice was decreased (Fig 5C). Then, we checked the levels of other antioxidants and found that the expression levels of antioxidant genes including SOD1, SOD2, GPX1, GSR, Txnrd1, GCL, Prdx3, HO1, catalase and NQO1 and of SOD2 and Prdx2 proteins were all down-regulated significantly in Cyp27b1^-/-^ mice (Fig 5D and 5E). Nrf2, which is a redox-sensitive transcription factor that activates many genes that encode antioxidant and detoxifying enzymes, was also down-regulated dramatically in Cyp27b1^-/-^ mice (Fig 5D). These data indicate that 1,25(OH)_2_D_3_ appears to function as an antioxidant to remove ROS and maintain intracellular redox balance.

## Discussion

A strong association between low level vitamin D and high risk of colon cancer and colonic inflammatory disease has been found in various epidemiological studies[1, 18, 20]. Animal studies have demonstrated that 1,25(OH)_2_D_3_ supplement could reduce cancer incidence in several rodent models of colon cancer[31–33] including Cyp27b1 knockout mice[10] which could develop severe colitis induced with DSS. It has been demonstrated that vitamin D has an anti-cancer effect in colon cancer; however, the underlying mechanism is still unclear. One of important mechanisms by which 1,25(OH)_2_D_3_ exerts its anti-neoplastic activity is to interfere with Wnt/β-catenin signaling[34, 35] which was abnormally activated in 93% of colon cancer[36]. Studies indicated that Wnt/β-catenin signaling was often influenced by inflammatory factors such as IL-1β and HGF[37, 38]. The other mechanism is to inhibit inflammation. Vitamin D supplementation has been linked to decreased circulating proinflammatory cytokines in patients with colorectal adenomas[39]. Meeker and his colleagues found that dietary vitamin D supplementation had a preventive effect on inflammation-associated colon cancer by lowering inflammatory cytokines induced in response to Hb in Smad3^-/-^ mice[33]. These suggest that 1,25(OH)_2_D_3_ might exert cancer preventive activity by interrupting the link between inflammation and cancer. Liu and his colleague had showed that 1,25(OH)_2_D_3_ deficient mice developed DSS-induced colitis[20]. In the present study, we discovered that Cyp27b1^-/-^ mice, fed on the rescue diet to normalize the serum levels of calcium and phosphorus, automatically underwent chronic colonic inflammation leading to weight loss, shortened colon length and inflammatory cell infiltration. These suggest that 1,25(OH)_2_D_3_ may act to protect against the onset of colitis.

Besides its role in anti-tumorigenesis, 1,25(OH)_2_D_3_ also has an anti-aging effect. Previous studies have shown that Cyp27b1^-/-^ mice displayed premature aging including growth retardation, osteoporosis, TMJ-OA, etc[23, 40]. This phenotype was thought to result from DNA damage induced senescence. Consistently, our current study also demonstrated that DNA damage markers 8-OHdG and γ-H2AX, and cellular senescence marker SA-β-gal were increased, suggesting that 1,25(OH)_2_D_3_ deficiency may result in increased DNA damage and senescence of colonic cells. Low serum vitamin D levels have been associated with an elevation in levels of 8-OHdG, a marker of oxidative DNA damage[41].1,25(OH)_2_D_3_ reduces the level of γ-H2AX and ATM activation after induction of DNA damage by H_2_O_2_ or by N-nitroso-NFigmethylurea[42, 43]. Our finding of increased 8-OHdG and γ-H2AX in colon of Cyp27b1^-/-^ mice further supports the notion that 1,25(OH)_2_D_3_ deficiency may induce oxidative stress to cause DNA damage ultimately leading to the senescence phenotype of the colonic cells.

Oxidative stress could induce DNA damage. 1,25(OH)_2_D_3_ has been reported to function as anti-oxidant. Treatment of several cell lines with 1,25(OH)_2_D_3_ has been shown to induce enzymes involved in protection against oxidative damage such as thioredoxin reductase1 (TXNRD1), the enzyme which maintains thioredoxin in the reduced state and limits intracellular oxidant stress[44]. 1,25(OH)_2_D_3_ was also observed to attenuate H_2_O_2_‐induced increase of γH2AX in lymphocytes[43]. Consistent with previous studies, our results showed that increased oxidative stress including higher levels of intracellular ROS and H_2_O_2_ in Cyp27b1-deficient colons which may result from down-regulation of some endogenous antioxidant genes, including SOD1, SOD2, Gpx1,GSR, Prdx3 and catalase. Interestingly, we also observed that expression of two anti-oxidants, Nrf2 and Klotho, was down-regulated significantly at mRNA level. Nrf2, nuclear factor erythroid 2-related factor 2, is a redox-sensitive transcription factor that activates many genes encoding antioxidant and detoxifying enzymes such as SOD1, SOD2, catalase, glutamate cysteine ligase (GCL), haemoxygenase 1 (HO1), NAD(P)H quinone oxidase 1 (NQO1), and peroxyredoxins (Prdx)[45]. Klotho, an anti-aging gene, was revealed to significantly enhance the expression of the thioredoxin/peroxiredoxin (Trx/Prx) system with the greatest effect on the induction of Prx-2 that acts to reduce ROS[46]. Klotho mutant mice (C3H) with a severe decline in cognition were also discovered to be associated with an increase in oxidative stress[40]. Coincidently, several studies have shown that expression of Nrf2 and Klotho was both regulated by 1,25(OH)_2_D_3_[24, 47–49], suggesting that 1,25(OH)_2_D_3_ acting in conjunction with Klotho and Nrf2 regulates expression of many of the antioxidant systems that prevent oxidative stress by removing ROS. Our data indicate that 1,25(OH)_2_D_3_ deficiency induced the increase in ROS formation may result from the reduction of antioxidant gene expression by down-regulating Nrf2 and Klotho.

We observed that DNA damage induced cell senescence as evidenced by increased SA-β-gal in Cyp27b1 deficient colon. The higher levels of SA-β-gal in colon of Cyp27b1^-/-^ mice suggest that this type of persistent DNA damage may accelerate the senescence process. Senescent cells with persistent DNA damage were accompanied by a striking increase in the secretion of more than 40 factors including a variety of proinflammatory cytokines (most notably IL-1ɑ, IL-1β, IL-6, and IL-8), chemokines (chemoattractants and macrophage inflammatoryproteins), growth factors (HGF, TGFβ, GM-CSF) and matrix-remodeling enzymes influencing their environment. This phenotype has been termed the “senescence-associated secretory phenotype”, or SASP[50]. As expected, we found that the gene expression levels of IL-1α, IL-1β, IL-6, IL-8, HGF1, MMP-3 and MMP-13 and the percentage of those factors positively stained cells were all increased significantly in colon from Cyp27b1^-/-^ mice compared to their wild-type littermates. We also found that NF-κB signaling was activated in Cyp27b1^-/-^ mice, which is consistent with a previous study showing that NF-κB signaling is the major signaling pathway for transcriptional regulation of SASP[51]. Many SASP factors were shown to have oncogenic properties, mostly by affecting the tumor microenvironment. For instances, irradiation of stromal cells could affect the invasive phenotype of pancreatic cancer through secreting HGF and other growth factors to modify tumor-stromal interactions[52]. IL‑6 and IL‑8 that are secreted from senescent cells could promote tumorigenesis in premalignant cells[25, 53]. IL-1was generated following activation of the inflammasome in senescent cells. IL-1ɑ is a key mediator of SASP activation and can control activation of IL-6 and IL-8[54]. MMP3 could promote tumorigenesis in neighboring premalignant epithelial cells through paracrine effects[55].

In summary, our current study demonstrated that 1,25(OH)_2_D_3_ deficien**t mice** display a chronic inflammation in colon tissue, which may result from increased oxidative stress and DNA damage, and subsequently, induced cell senescence and overproduction of senescence-associated secretory factors including some pro-inflammatory factors and matrix metalloproteinases. Therefore, the findings from this study disclose a potential mechanism of 1,25(OH)_2_D_3_ in protection against colitis and colon cancer.

## Supporting Information

S1 Table Primary antibodies used in Immunohistochemistry. (DOC)

S2 Table Primary antibodies used in Western Blotting. (DOC)

## Abbreviations

SASP: Senescence-associated secretory phenotype
VDR: vitamin D receptor
TMJ: temporomandibular joint
OA: osteoarthritis
PTH: parathyroid hormone
DCFDA: diacetyldichlorofluorescein
