# Supplementary material for: 1,25(OH)2D3 Deficiency Induces Colon Inflammation via Secretion of Senescence-Associated Inflammatory Cytokines
Source: PLoS One. 2016 Jan 20;11(1):e0146426. doi: 10.1371/journal.pone.0146426 (PMC4720393; doi:10.1371/journal.pone.0146426)
Supplement: S1 Table — (DOC) [file pone.0146426.s001.doc]

**S1 Table. Primary antibodies used in Immunohistochemistry**

| **Primary antibody name** | **Company** | **Dilution** |
| --- | --- | --- |
| CD3 | AbD Serotec,UK | 1:200 |
| F4/80 | AbD Serotec,UK | 1:200 |
| 8-OHdG | Abcam, MA | 1:200 |
| Phospho-Histone H2AX | Cell Signaling Technology, Danvers, MA | 1:400 |
| IL-1α | Santa Cruz, CA | 1:200 |
| IL-6 | Santa Cruz, CA | 1:200 |
| IL-8 | Abcam, MA | 1:200 |
| HGF1 | Abcam, MA | 1:200 |
| MMP-3 | Bioworld Technology, St. Louis Park, MN | 1:200 |
