# Supplementary material for: 1,25(OH)2D3 Deficiency Induces Colon Inflammation via Secretion of Senescence-Associated Inflammatory Cytokines
Source: PLoS One. 2016 Jan 20;11(1):e0146426. doi: 10.1371/journal.pone.0146426 (PMC4720393; doi:10.1371/journal.pone.0146426)
Supplement: S2 Table — (DOC) [file pone.0146426.s002.doc]

S2 Table. Primary antibodies used in Western Blotting.

| **Primary antibody name** | **Company** | **Dilution** |
| --- | --- | --- |
| SOD2 | Abcam, MA | 1:2000 |
| NF-κB-p105/p50 | Cell Signaling Technology, Danvers, MA | 1:1000 |
| Phospho-Histone H2AX | Cell Signaling Technology, Danvers, MA | 1:1000 |
| β-actin | Cell Signaling Technology, Danvers, MA | 1:2000 |
